# Computational and experimental identification of putative αTAT1 modulators: implications for nervous system function

**DOI:** 10.3389/fphar.2025.1654114

**Published:** 2025-10-07

**Authors:** Oksana Rybachuk, Alexey Rayevsky, Mariia Styhylias, Dariia Samofalova, Elijah Bulgakov, Maxym Platonov, Yaroslav Blume, Pavel Karpov

**Affiliations:** ^1^ Bogomoletz Institute of Physiology, National Academy of Sciences of Ukraine, Kyiv, Ukraine; ^2^ Institute of Genetic and Regenerative Medicine, M. D. Strazhesko National Scientific Center of Cardiology, Clinical and Regenerative Medicine, National Academy of Medical Sciences of Ukraine, Kyiv, Ukraine; ^3^ Institute of Food Biotechnology and Genomics, National Academy of Sciences of Ukraine, Kyiv, Ukraine; ^4^ Institute of Molecular Biology and Genetics, National Academy of Sciences of Ukraine, Kyiv, Ukraine; ^5^ Enamine Ltd., Kyiv, Ukraine

**Keywords:** αTAT1, microtubule, docking, pharmacophore, neural stem cells

## Abstract

**Introduction:**

The project’s primary objective is to understand how enzymes responsible for post-translational modifications (PTMs) of microtubule elements influence ion channels in excitatory peripheral nervous system (PNS) cells, and to subsequently identify potential pharmacological agents that can act upon these molecular targets. Having identified HDAC6 and αTAT1 as the most relevant proteins for further study, we focused on αTAT1. αTAT1 is the sole mammalian enzyme known to acetylate microtubules, a process associated with regulating their dynamics and protecting long-lived microtubules from mechanical stress. Additionally, αTAT1 plays a role in nuclear processes such as DNA replication, cell migration, and axonal transport. Given the importance of αTAT1 in cytoskeletal homeostasis and the lack of known effectors, our research focused on this enzyme.

**Methods:**

We designed a corresponding pharmacophore model hypothesis of ligand-dependent inhibition for αTAT1 and performed search for potential αTAT1 modulators using molecular interaction analysis and existing crystal structures. Confocal microscopy was used to assess cytoskeletal changes in a neural stem cell line following experimental treatment.

**Results:**

The activity of identified hits was confirmed via confocal microscopy. This allowed for further tuning of our screening models and generation of novel subsets of compounds.

## Introduction

Microtubules (MTs), the primary components of the eukaryotic cytoskeleton, are formed by the polymerization of α- and β-tubulin heterodimers. The diverse tubulin isotypes and a variety of post-translational modifications (PTMs) tightly regulate MT properties and functions, giving rise to the “tubulin code” (Janke and Magiera, 2020). These PTMs can be reversible, such as phosphorylation, or likely irreversible, for instance, nitration (Iuzzolino et al., 2024; Sánchez-Fernández et al., 2023).

Phosphorylation is considered the most common and well-studied of all known microtubule (MT) post-translational modifications (PTMs) (Janke and Magiera, 2020; Fu et al., 2015; Samofalova et al., 2019). As a result, tubulin phosphorylation can almost completely inhibit microtubule polymerization (U et al., 2020). In addition to phosphorylation, acetylation is a critical tubulin PTM that influences microtubule polymerization levels and has an indirect impact on autophagy processes (Coombes et al., 2020; Aguilar et al., 2014; Wong et al., 2014). This effect was subsequently confirmed experimentally, demonstrating significant alterations in tubulin heterodimer stability (Sardar et al., 2012). This finding strongly supports the link between α-tubulin acetylation, the formation of stable microtubules, and their protection from mechanical ageing (Goswami et al., 2010). Although a major part of the components involved in this process has already been described in detail, the mechanism of microtubule stabilization due to this modification has not yet been elucidated and requires further research (Dybkova et al., 2014; Rayevsky et al., 2023; Rayevsky et al., 2025; Rayevsky et al., 2019).

Rearrangements within α-tubulin’s secondary structure are crucial for the dynamic reorganization of microtubules. Numerous studies have highlighted the role of α-tubulin N-acetyltransferase (αTAT1), an enzyme typically found in animal cells, in acetylating α-tubulin at the Lys40 position (Wong et al., 2014; Rayevsky, et al., 2025; Huang et al., 2017). The reversible process is provided by HDA6/12 deacetylases (U et al., 2020; Ferreira de Freitas et al., 2018; Stykhylias et al., 2024). Adding to the complexity, αTAT1 acetylates Lys40 and Lys60 on the luminal side of microtubules, raising the question of how the enzyme accesses this site (Rakhshani et al., 2019; Liu et al., 2015). Studies have demonstrated that induced dysfunction of αTAT1 results in a substantial loss of mechanical sensitivity in peripheral nervous system cells. Intriguingly, despite the significant evolutionary conservation of tubulins and confirmed microtubule acetylation in plants, neither αTAT1 nor proteins exhibiting comparable predicted function have been identified in the plant kingdom (Friedmann et al., 2012). At the same time, MYST and p300-like enzymes can acetylate plant proteins (Han et al., 2007; Rayevsky et al., 2016).

Therefore, lysine acetylation is a key post-translational modification (PTM) governing microtubule structure and interactions with tau and other microtubule-associated proteins involved in Alzheimer’s disease. However, αTAT1’s role extends beyond microtubules; it can participate in multi-protein complexes and modify unidentified substrates, crucial in DNA replication and repair (Wu et al., 2018). αTAT1 exhibited non-enzymatic activities in cell migration, non-axonal intracellular transport and non-neuronal diseases (Topalidou et al., 2012; Dan et al., 2018). Also, too little is known about the significance of ATAT1 acetyltransferase activity in cancer development and any details of the underlying process. Due to the undoubted and important role of the enzyme and the lack of published data on modulators of αTAT1 activity, this area of research is promising in terms of fundamental knowledge and practical application of the results. Our task was to develop models for searching commercial databases and further confirm the activity of hits by analyzing the state of the cytoskeleton of neuronal cells using confocal microscopy.

## Methods and materials

### Molecular docking and pharmacophore search

The human αTAT1 sequence was taken from the UniProtKB database (Q5SQI0) and analyzed using the internal services in an attempt to find any similar proteins or even complexes in the Protein Data Bank (UniProt Consortium, 2025; Zardecki et al., 2022). Since only the catalytic domains of the target protein were found, we used two complexes with acetyl-coenzyme A and a bisubstrate analogue (PDB ID: 4G4 and 4PK3) as a starting point for the binding mode assessment. To describe the extended and vaste binding pocket of 4PK3, we employed both structural methods (PyMOL plugin, CavitOmiX, PLIP) and a ligand-based site mapping method (LigPlot with LigandScout 4.4) (Wolber et al., 2006; Wolber and Langer, 2005; La et al., 2011; Adasme et al., 2021). Consequently, we mapped all potential ligand-protein interactions within the binding cavity, including donors and acceptors, to determine their reactivity and identify crucial residues and atoms.

The centroids for subsequent descriptors were then determined based on the coordinates of CavitOmiX innophores that filled the pocket, selected according to the canonical distances of their respective interaction types. The remaining pseudoatoms of the cloud were removed. The resulting structure (protein fragment + remaining probes) was saved in the *.pdb format and using the built-in tool final refinement of the model was completed in the LigandScout program.

An important aspect was that LigandScout allowed the built model to be saved in the *.pmz format, which is compatible with the Pharmit online service (https://pharmit.csb.pitt.edu/search.html) and connected to the Enamine compound library, which was the specific library that needed to be screened. During the final system setup, the properties of the features were defined as follows: their diameters were set (R = 1), interaction vectors were adjusted according to the donors and acceptors of the target site, as well as according to the centroids of cyclic fragments of aromatic amino acids, allowing for parallel/perpendicular stacking (π-π). For cyclic interactions, the features allowed for π-π and/or hydrophobic interactions. Ultimately, the ligand docking model in the binding site was constructed based on three anchors formed by assigning hydrophobic regions, four hydrogen-bonding atoms, and one steric constraint (corresponding to a negatively charged group) in the region of the acetyl and diphosphate sites (one interaction with R158 or K165 in the acetyl site is required). Screening results were ranked according to their “Score.” The Score threshold was defined as the minimum Score value found in the control group compounds.

The control group of compounds was additionally processed with a molecular docking procedure performed in the CCDC Gold 5.3 program (www.ccdc.cam.ac.uk), which works based on a genetic algorithm for creating new ligand conformations. Hydrogens have been added to the protein structure by *Hermes*. Experimentally solved conformation of the protonated ligand was used to define interactions and binding area. Radius of the binding site area (5Å) for the docking was defined selecting the phosphate group of the substrate in the crystal structure (4PK3). Initially we supposed to apply H-bond constraints to the backbone atoms of amino acids Ile126, Gly134, Gly136, but shifted our attention at the interactions with amino acids Arg132 and His170, which were set as H-bond constraints. Residue Gln131 was set as flexible to increase as we suggested it can find more favorable interaction with novel ligands. Then we defined hydrophobic area between Phe124 and Phe166 as a hydrophobic region constrain. The docking process utilized standard parameters, including a population size of 100, a sample size of 1.1, 10 islands, and 100,000 genetic operations. Docking maps were created in a rectangular box with a 0.5 Å grid spacing, centered on the predicted ligand binding site. The fitness function settings included a ChemPLP, as a first sieve to pass with further re-scoring by ASP function (the Astex Statistical Potential). Finally, the outcome of screened compounds was analyzed with Datawarrior to predict its ADME parameters (López-López et al., 2019). All the computational procedures were utilised in our virtual laboratory, CSLabGrid (Karpov et al., 2015; Karpov et al., 2016).

### Isolation of the neural stem cells and viability assay

Animals were used by the protocols approved by the Animal Care and Use Committee at Bogomoletz Institute of Physiology (Kyiv, Ukraine) and the Law of Ukraine on the protection of experimental animals (N3447-IV, 21.02.2006). The method of isolation we described previously (Rybachuk et al., 2019). Briefly, hippocampal NSCs from 16–17 dpc FVB-wt mouse embryos (n = 12) were mechanically dissociated in Neurobasal medium (Gibco, United States) under aseptic conditions. The isolated cell suspension was filtered using a 40 μm nylon cell strainer. (Falcon, United States). The NSPC fraction was purified by centrifugation in a 22% Percoll density gradient at 450 g for 10 min (GE Healthcare BioScience, Sweden). Cell viability was determined by flow cytometry based on 7-aminoactinomycin D (7-AAD) incorporation and analyzed using a BD FACSAria cell sorter (Becton Dickinson, United States) and BD FACSDiva software version 6.2.1 (Becton Dickinson, United States).

### Culturing of neural stem cells in the medium supplemented with different compounds (C1, C2, C3, C4, and C5)

NSCs were plated on Matrigel-coated coverslips (BD Biosciences, UK) at a density of 5 × 10^4^ cells per well in a 24-well plate and incubated under standard culture conditions (5% CO_2_, 37 °C) for 48 h. The standard culture medium was Neurobasal medium (Gibco, United States) supplemented with 20 ng/mL bFGF (Sigma, United States), 2% B27 supplement (Invitrogen, United States), 1 mM sodium pyruvate (Invitrogen, United States), 1 mM N-acetyl-L-cysteine (Sigma, United States), 1% L-glutamine (HyClone, United States) and 1% penicillin-streptomycin (Invitrogen, United States).

After 48 h, NSCs were divided into 22 experimental groups and cultured for another 48 h: (a) standard culture medium; (b) standard culture medium + 10 μM C1; (c) standard culture medium + 100 μM C1; (d) standard culture medium + 10 μM C2; (e) standard culture medium + 100 μM C2; (f) standard culture medium + 10 μM C3; (g) standard culture medium + 100 μM C3; (h) standard culture medium + 10 μM C4; (i) standard culture medium + 100 μM C4; (j) standard culture medium + 10 μM C5; (k) standard culture medium + 100 μM C5; (L) standard culture medium deprived of bFGF; (m) standard culture medium deprived of bFGF + 10 μM C1; (n) standard culture medium deprived of bFGF + 100 μM C1; (o) standard culture medium deprived of bFGF + 10 μM C2; (p) standard culture medium deprived of bFGF + 100 μM C2; (q) standard culture medium deprived of bFGF + 10 μM C3; (r) standard culture medium deprived of bFGF + 100 μM C3; (s) standard culture medium deprived of bFGF + 10 μM C4; (t) standard culture medium deprived of bFGF + 100 μM C4; (u) standard culture medium deprived of bFGF + 10 μM C5; (v) standard culture medium deprived of bFGF + 100 µM C5.

The total duration of cultivation after seeding was 4 days. The growth of NSCs and the formation of primary adhesive spheres (neurospheres) during cultivation under different conditions were observed using an inverted microscope (Olympus IX 71, Japan) with relief phase contrast.

### Immunocytochemical analysis of cultured NSCs

NSC cultures were fixed in 4% paraformaldehyde for 30 min and then washed with a washing buffer (0.1 M PBS). Blocking was carried out for at least 2 h using a blocking solution for permeabilization (washing buffer supplemented with 0.3% Triton X-100 and 0.5% BSA). The NSCs were incubated overnight at 4 °C in blocking solution in the presence of primary antibodies at the concentrations recommended by the manufacturer. To identify NSCs, proliferating cells and differentiated NSCs into neurons, double immunostaining was performed using antibodies against Nestin (1:100, Santa Cruz Biotecnology, United States), Ki-67 (1:200, Abcam, UK), ɑ-tubulin (1:200, Abcam, UK), β(III)-tubulin (1:700, Sigma, Germany) and MAP-2 (1:2000, Abcam, UK). Nestin expression was adopted as a marker for neural stem and precursor cells (Jan and Kotov, 2007; Xiong et al., 2014); β-tubulin (III) is a specific marker for neuronal lineages (Xiong et al., 2014).

After that, the cells were washed with the washing buffer 3 times for 15 min and then incubated in the presence of secondary antibodies for at least 2 h at room temperature. Secondary antibodies were: anti-chicken Alexa Fluor 488 (1:1000, Invitrogen, United States), anti-mouse Alexa Fluor 555 (1:1000, Invitrogen, United States) and anti-rabbit Alexa Fluor 647 (1:1000, Invitrogen, United States). Images were taken with a laser scanning confocal microscope FluoView^TM^FV1000 (Olympus, Japan).

### Statistical analysis

Statistical analyses were performed using statistical software (version 5, StatSoft, Tulsa). The results are presented as mean ± standard error of the mean (SEM). Chemical effects on cells were determined using a one-way analysis of variance (ANOVA) with Tukey *post hoc* HSD. The Kruskal-Wallis test was applied to compare the number of formed primary neurospheres under different cultivation conditions. The difference between means was considered significant at P < 0.05.

## Results

### In silico search for potential αTAT effectors

Our preliminary analysis of the αTAT1 structure (4PK3, 1.35 Å) indicated that the active site features an extended, bulky binding pocket. This pocket exhibits minimal ligand-protein adaptation features, likely due to its size and the nature of the substrate. Furthermore, the structure accuracy analysis yielded a global QMEANDisCo index of 0.83 ± 0.05 (Studer et al., 2020), while Ramachandran plot analysis showed that 94.82% of amino acid residues were in the allowed regions.

**FIGURE 1:**
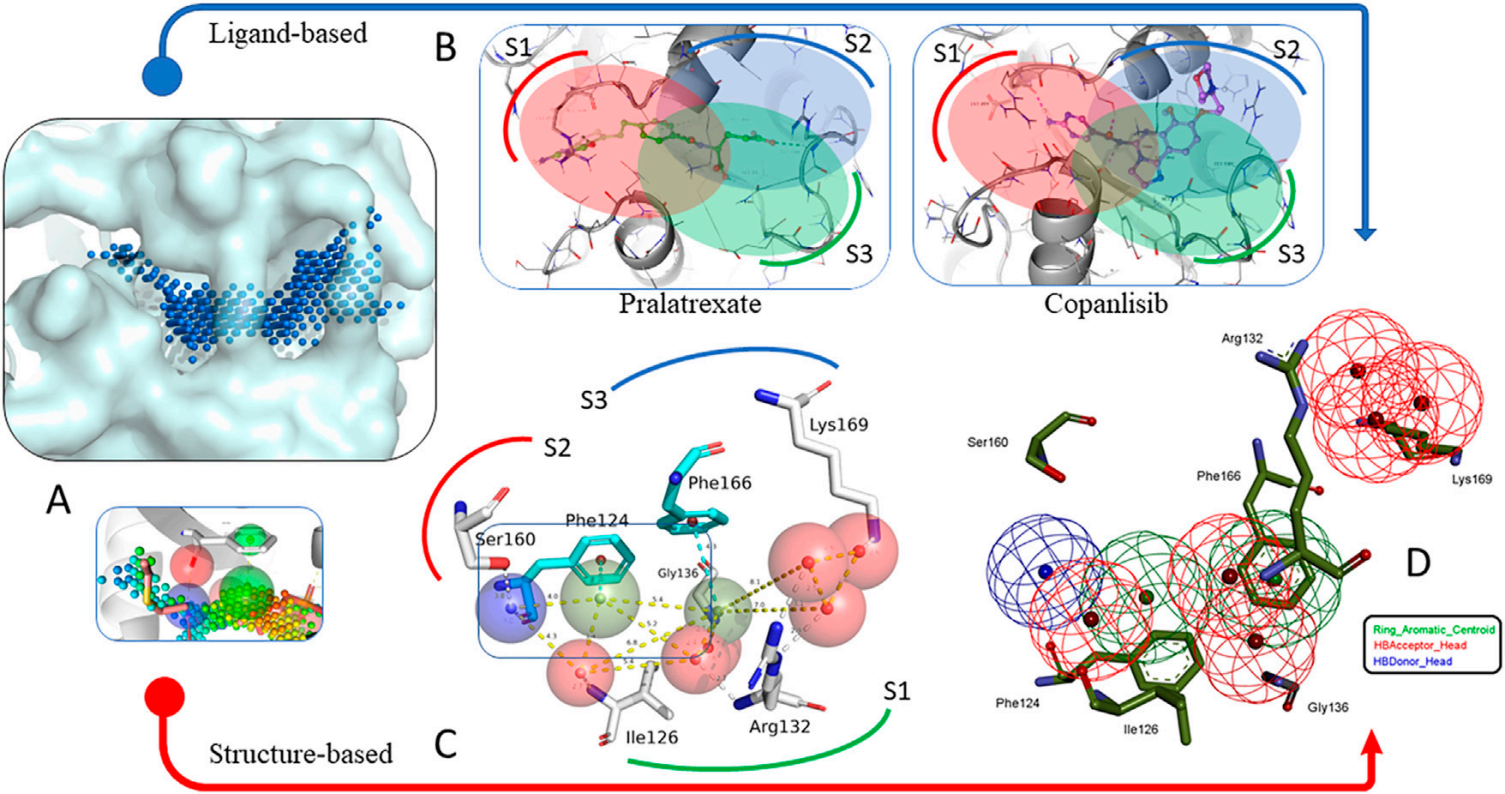
Two strategies were developed for the fine screening model generation. **(A)** Binding site mapping with ionophores (charged probes). Coloring scheme reflects positions of hydrogen bond donors (blue) and acceptors (red), as well as positions of possible cyclic fragments (green). **(B)** Results of molecular docking for the best of potential αTAT effectors proposed by Hsu et al., 2021. The outcome didn’t match the expected poses and interaction distribution (outlined with ovals). **(C)** Identification of coordinates of the centroids and the volume of individual pharmacophore features (distance-optimal pseudoatoms). **(D)** The resulting Pharmacophore model used for the screening.

We started by testing information about the amino acid composition and surface features of the pocket. The corresponding centroids of the future descriptors were determined among the CavitOmiX innophores that filled the target site pocket and were selected based on the canonical distances of the corresponding interaction types (Figure 1). It was separately found that, taking into account the distance of 4 Å, 6 aromatic amino acids can be involved in ligand binding, namely: Phe124, Tyr125, Phe166, His135, Phe166 and His170.

Further structural analysis of topology and conformational mobility determined that only Phe124 and Phe166 can form potential π-π interactions with the ligand. Thus, a final skeleton of the future pharmacophore was constructed with interaction combinations for future searches. The final model consisted of nine descriptors: 6 hydrogen bond acceptors, 1 hydrogen bond donor, and 2 cyclic fragment markers.

Simultaneously, we conducted docking studies on 10 candidate αTAT1 inhibitors identified by Hsu et al. (2021). According to the CCDC GOLD FitnessScore, 7 of these compounds exhibited a predicted binding affinity for the target site stronger than that of the natural substrate (acetyl-CoA from 4PK3), yet their binding orientations showed no common trends. At the same time, two compounds (Ceftolozane and Oftasceine) were designated as non-cognate ligands, showing the absence of sufficient interactions. Another, Mangafodipir, was hard to dock and finally interpreted its poses, because of a complex metal-chelating structure (Table 1).

**TABLE 1:** Docking results for the control group and selected screening hits.

Source	4PK3	Cefonicid	Ceftolozane	Copanlisib	FolicAcid
ChemPLP	49.48	58.53	51.95	57.74	65.86
ASP	−58.04	−61.08	−54.95	−60.37	−68.33
Source	Hesperidin	Pralatrexate	Methotrexate	Oftasceine	Pemetrexed
ChemPLP	58.2	71.65	66.58	−16.58	67.92
ASP	−61.25	−74.17	−69.02	11.49	−70.53

**FIGURE 2:**
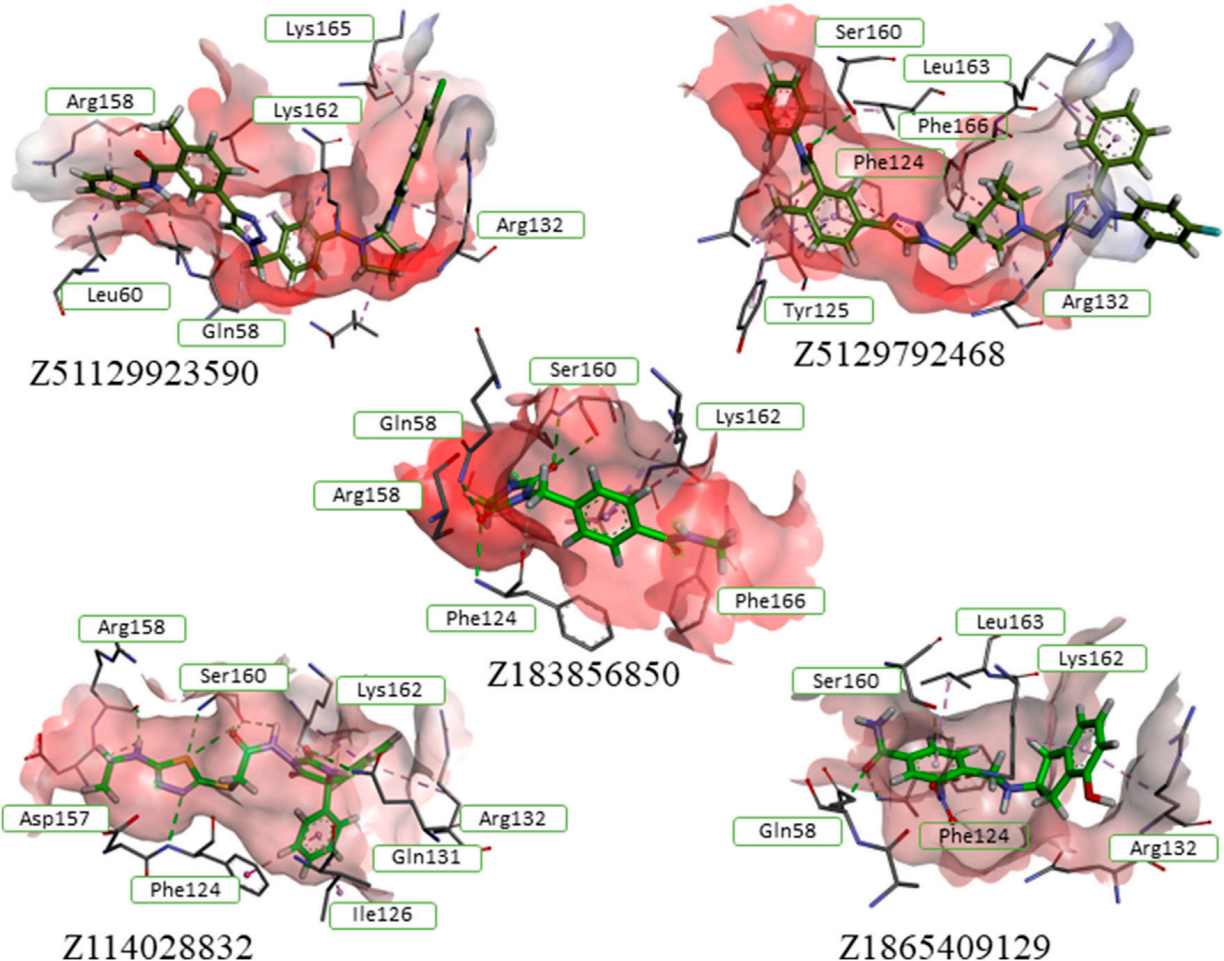
Results of the analysis of the binding forces for the top-performing compounds, based on ChemPLP and ASP evaluation functions.

Having analyzed the rates of interactions between different residues and the substrate (crystal complexes of αTAT1) or these 10 compounds, we identified overlapping functional groups in the site of interest. Together with our previous suggestions based on binding site deconstruction, we selected all relevant features in a structure-based protocol to generate target-oriented prediction of potential interactions with novel ligands other than the cognate substrate. From these, we divided the total pool of contacts between the protein and the ligand into three groups of interactions different from those declared by Hsu et al. (2021). First group includes amino acid residues His133, Arg137, Gly134, Gly136, Ser160, Phe124 and Ile126 form hydrogen bonds with keto-, phosphate-, sulfo- and amino groups of the ligand. A second set of residues, Lys162, Asp123, and Tyr125, can interact with the ligand via weak hydrogen bonds involving the thiazole heterocycle and keto groups. As an alternative interaction mode, a third group, Arg132 and Lys169, can form charged contacts with the ligand’s heterocycles and phosphate groups. Using the PharmIT web service (https://pharmit.csb.pitt.edu/search.html), a scan of the publicly available online ligand repositories: ChEMBL DB, ZINC DB, and PubChem was performed. Using the full pharmacophore, 10 hits were found in the ChEMBL database and 78 hits in the ZINC database (Figure 2).

**FIGURE 3:**
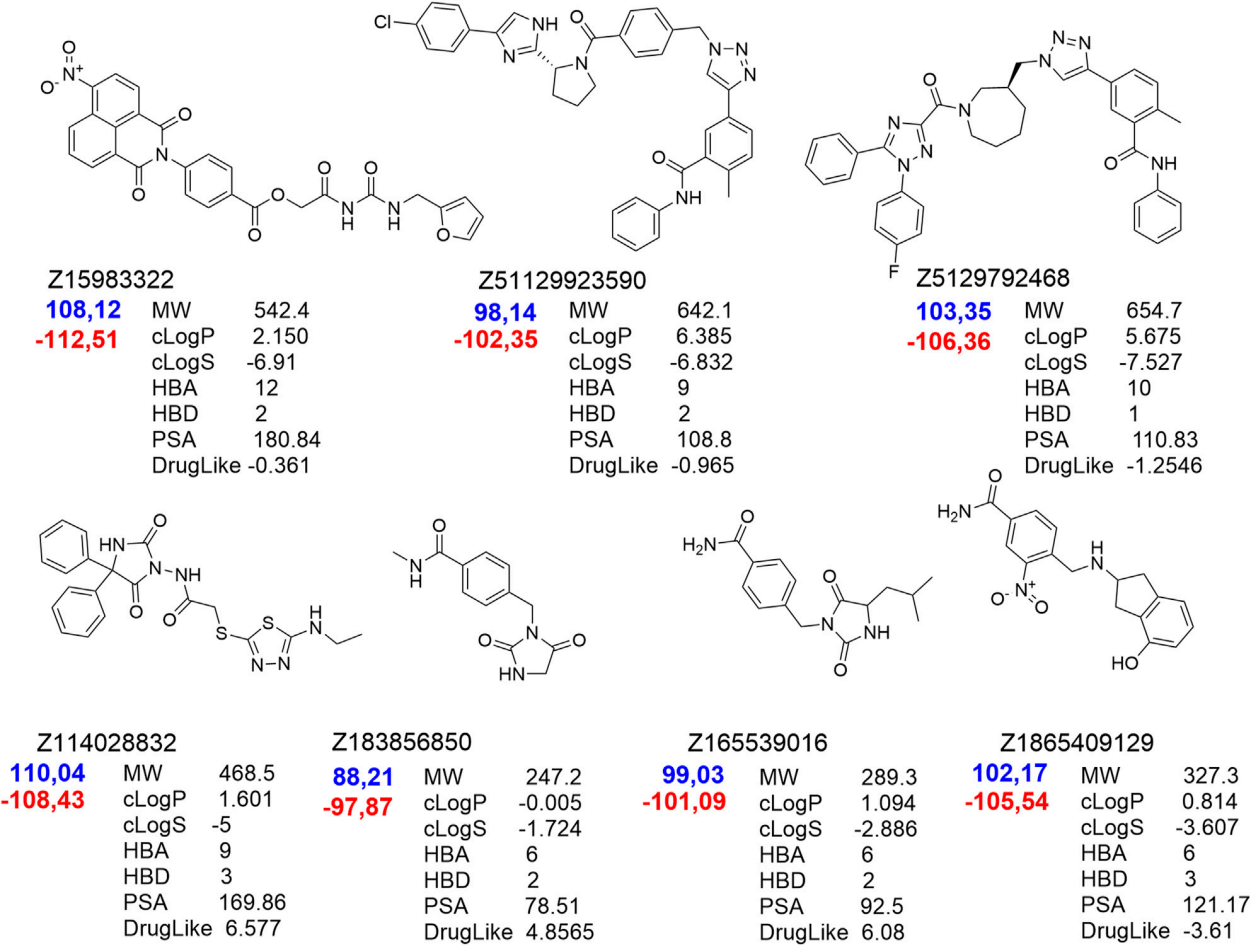
Hit compounds from the ZINC DB showed best scores in the final docking of the PharmIT search results. Values of the scoring functions are represented in colors - Score (red labels) and S(PLP) (dark blue labels).

Molecular re-docking in the CCDC GOLD program using a combination of the ChemPLP and ASP fitness functions determined only 7 leaders fitting the target site without clash conflicts and significant indicators of affinity and quality of the complexes. It should be noted that selected ligands had interaction indices higher than that of the best compound from the control group – Pralatrexate (Figure 3). These 7 compounds were analyzed with DataWarrior to predict ADME parameters and show distribution of drug-likeness properties (MW – molecular weight, HBA/HBD – acceptors/donors, cLogP/cLogS – solubilities, PSA – polar surface area). Trying to reach balance between values of drug-likeness, molecular weight and docking scores we selected only five compounds ready to be assessed in cells.

### Experimental confirmation of the aTAT1-targeting

**FIGURE 4:**
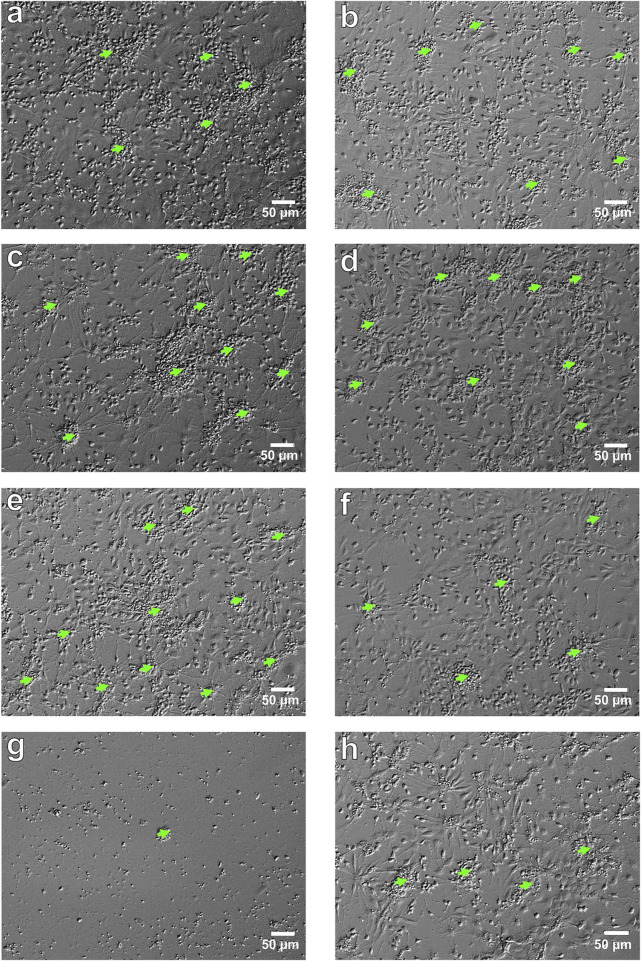
NSCs *in vitro*. Micrograph of NSCs in various culture conditions: **(a)** control, NSCs culturing with bFGF (NSCs + bFGF); **(b)** control, NSCs culturing without bFGF (NSCs); **(c)** NSCs + bFGF+10 µM C2 (48 h); **(d)** NSCs+100 µM C2 (48 h); **(e)** NSCs + bFGF+100 µM C2 (48 h); **(f)** NSCs+10 µM C4 (48 h); **(g)** NSCs + bFGF+10 µM C5 (48 h); **(h)** NSCs+100 µM C4 (48 h). Relief contrast inverted microscope; light green arrows–primary adhesive neurospheres; scale bars = 50 µm.

**FIGURE 5:**
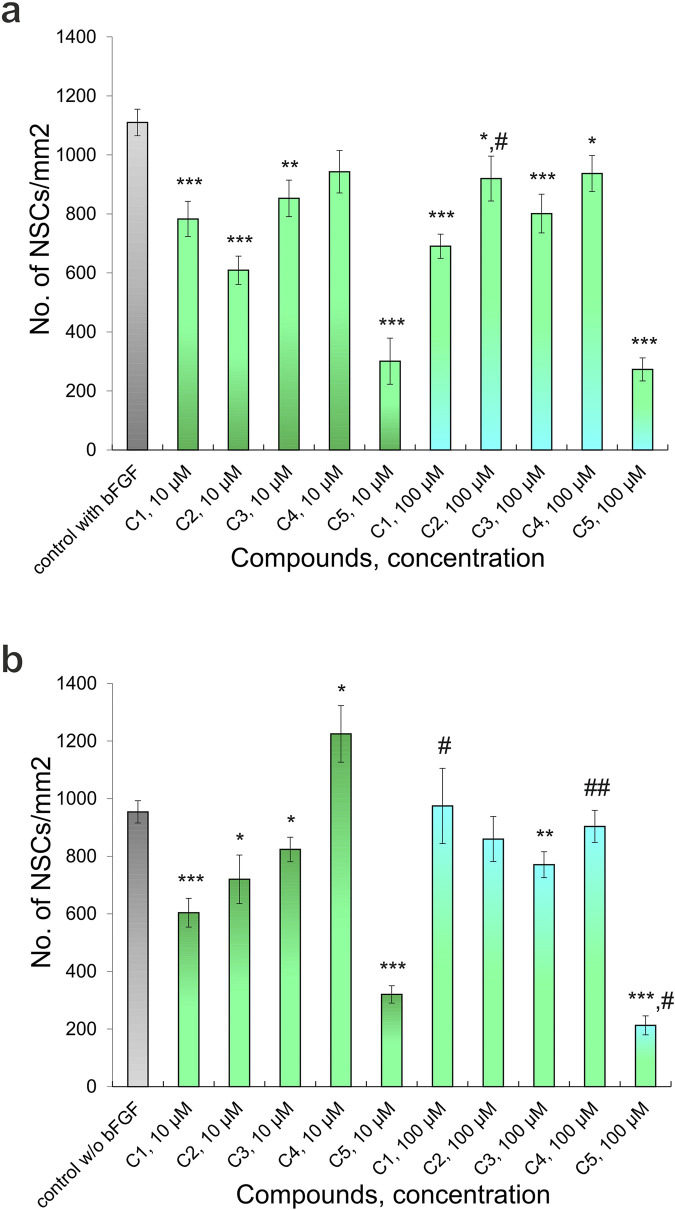
Effect of different concentrations of Compounds (C1-C5) on the NSCs (48 h culturing): **(a)** culturing with added bFGF, **(b)** culturing without added bFGF. P-values derived from the Student’s two-tailed t-test, the results are shown as mean ± SEM; *p < 0.05, **p < 0.01 and ***p < 0.001 *versus* control with bFGF **(a)** and *versus* control without bFGF **(b)**; #p < 0.05 and ##p < 0.01 *versus* different concentrations of one’s compound.

According to the results of testing the viability of NSCs, it was found that the percentage of live cells in the suspension was not less than 94.8% ± 0.3%, which is a high indicator of the viability of freshly isolated NSCs. When culturing NSCs under standard conditions (with the addition of bFGF), the number of cells was 1109.96 ± 44.82 (n = 47) per mm2, and when 10 μM of C1, C2, C3, C4, and C5 (Z1865409129, Z114028832, Z165539016, Z183856850, and Z15983322, respectively) were added to the culture medium, the number of NSCs significantly decreased to 782.85 ± 59.77 (n = 16) (p < 0.001), 609.12 ± 48.01 (n = 17) (p < 0.001), 852.72 ± 62.01 (n = 17) (p < 0.01) and 300.86 ± 78.28 (n = 15) (p < 0.001) per mm^2^, respectively. (Figures 4a,c,g, 5a) while the addition of the same concentration of C4 did not lead to a significant and reliable change in the number of cells - 943.01 ± 72.14 (n = 18) (Figure 5a)- 943.01 ± 72.14 (n = 18) (Figure 5a). At the same time, increasing the concentration of C1-C5 to 100 μM in the cultivation medium led to significant changes in the number of NSCs, namely, their decrease: 690.56 ± 40.92 (n = 16) (p < 0.001), 919.92 ± 76.04 (n = 17) (p < 0.05), 801.08 ± 65.51 (n = 17) (p < 0.001), 937.21 ± 61.03 (n = 18) (p < 0.05) and 272.83 ± 39.17 (n = 15) (p < 0.001) per mm^2^, respectively (Figures 4e, 5a). It should be noted that the presence of C2 in the culture medium at a concentration of 100 μM contributes to the greater cellularity (p < 0.01) compared to NSCs cultured at a 10 μM concentration of this substance (Figures 4c,e, 5a).

In cultures grown without bFGF, the number of NSCs was 954.06 ± 38.52 per mm2 (n = 35), a significant decrease compared to control cultures with bFGF (p < 0.01). The addition of 10 μM of compounds C1, C2, C3, and C5 further significantly reduced the number of NSCs to 603.83 ± 49.86 (n = 16, p < 0.001), 720.01 ± 84.21 (n = 15, p < 0.05), 823.74 ± 42.52 (n = 19, p < 0.05), and 320.17 ± 30.21 (n = 14, p < 0.001) per mm^2^, respectively. (Figure 4b, 5b); however, addition of C4 at this concentration significantly increased the number of NSCs to 1224.93 ± 98.27 (n = 17) (p < 0.05) (Figures 4f, 5b). Adding 100 μM concentrations of C1, C2, and C4 resulted in no significant quantitative changes in the NSC culture: 974.54 ± 130.51 (n = 18), 859.60 ± 78.19 (n = 16), 903.59 ± 55.72 (n = 18) per mm^2^, respectively (Figures 4d,g,h, 5b). However, when C3 and C5 were added, the number of NSCs under the above cultivation conditions significantly decreased: 770.57 ± 45.07 (n = 17) (p < 0.01) і 212.78 ± 32.76 (n = 16) (p < 0.001) per mm^2^, respectively (Figure 5b). Intriguingly, in the absence of bFGF, while 10 μM C1 significantly decreased NSC numbers (as shown previously), increasing the C1 concentration to 100 μM resulted in a significant *increase* (p < 0.05) in cell number compared to the lower dose (Figure 5b). Similarly, in the absence of bFGF, C4 and C5 significantly reduced the number of NSCs (p < 0.01 and p < 0.05, respectively) (Figures 4f,h, 5b). Notably, C5 consistently caused a significant decrease in NSC numbers under all tested conditions (48 h), with only single cells observed in some wells (Figures 4g, 5a,b). These findings collectively indicate a cytotoxic effect of C5 on NSCs.

**FIGURE 6:**
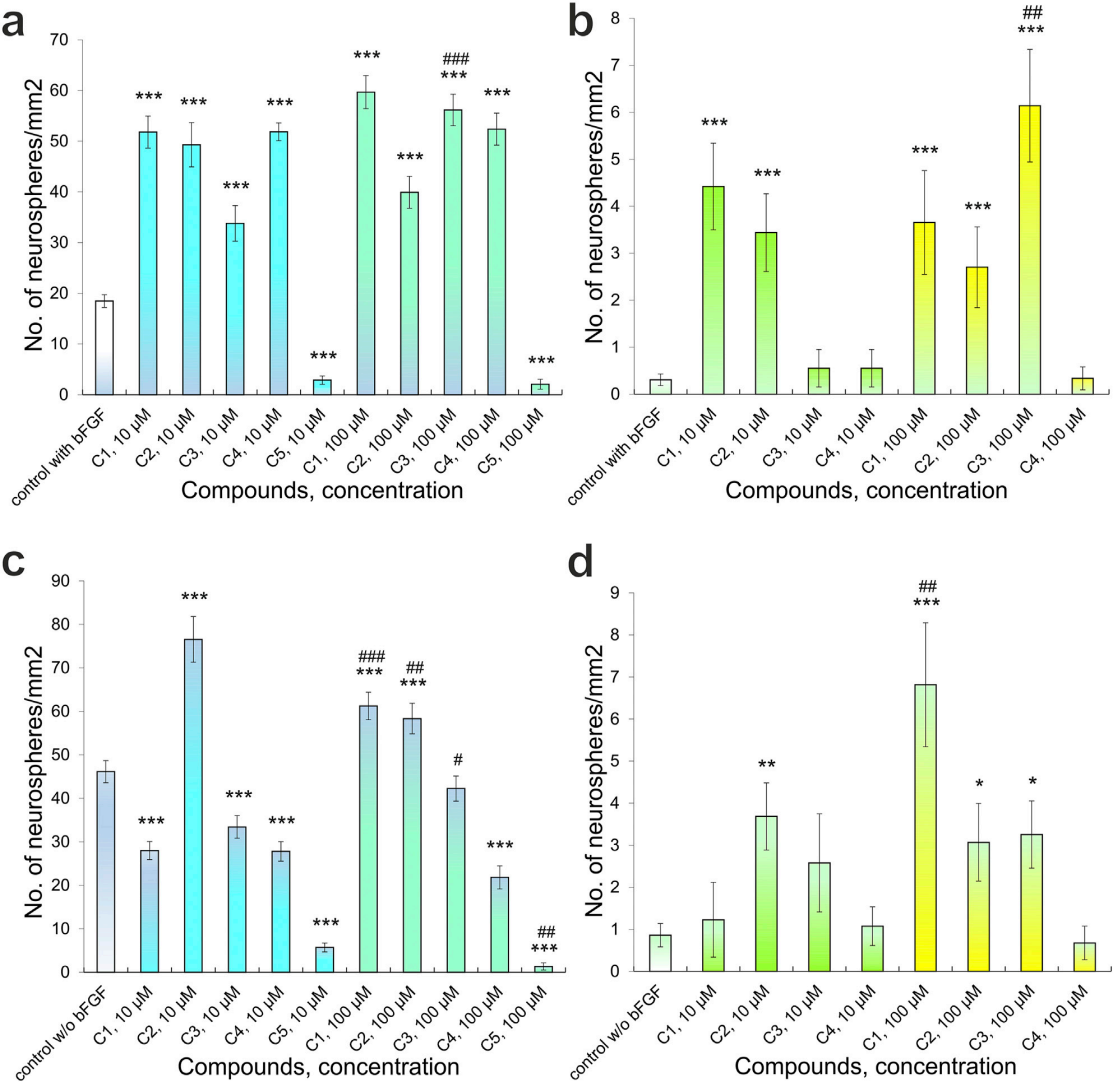
Quantitative analysis of primary adhesive neurospheres that form in the presence of Compounds (C1-C5): **(a)** the number of neurospheres < 50 µm in diameter, culture conditions with bFGF; **(b)** the number of neurospheres > 50 µm in diameter, culture conditions with bFGF; **(c)** the number of neurospheres < 50 µm in diameter, culture conditions without bFGF; **(d)** the number of neurospheres > 50 µm in diameter, culture conditions without bFGF. P-values derived from the Kruskal-Wallis test, the results are shown as mean ± SEM; *p < 0.05, **p < 0.01 and ***p < 0.001 *versus* control with bFGF **(a,b)** and *versus* control without bFGF **(c,d)**; #p < 0.05, ##p < 0.01 and ##p < 0.001 *versus* different concentrations of one’s compound.

Cultivation conditions influenced the formation of primary adhesive neurospheres. Specifically, in control cultures receiving bFGF supplementation, we observed the formation of 18.48 ± 1.26 neurospheres with a diameter less than 50 μm and 0.31 ± 0.12 neurospheres with a diameter greater than 50 μm per mm^2^ (n = 47) (Figures 4a,b, 6a,b). When 10 μM and 100 μM C1-C4 were added to the culture medium, the number of neurospheres with a diameter of <50 μm significantly increased: 51.79 ± 3.16 (n = 16) (p < 0.001), 49.30 ± 4.36 (n = 17) (p < 0.001), 33.77 ± 3.53 (n = 17) (p < 0.001), 51.85 ± 1.75 (n = 18) (p < 0.001) and 59.68 ± 3.25 (n = 16) (p < 0.001), 39.91 ± 3.16 (n = 17) (p < 0.001), 56.15 ± 3.10 (n = 17) (p < 0.001), 52.37 ± 3.16 (n = 18) (p < 0.001) per mm^2^ respectively (Figures 4c,e, 6a). However, the addition of C5 at concentrations of 10 μM and 100 μM significantly reduced neurosphere formation to 2.85 ± 0.83 (n = 15, p < 0.001) and 2.06 ± 0.98 (n = 15, p < 0.001) per mm^2^, respectively (Figure 6a). Similarly, varying concentrations of C3 also significantly impacted neurosphere formation (p < 0.001); specifically, increasing C3 concentrations led to a substantially greater number of neurospheres with a diameter <50 μm (Figure 6a).

The addition of compounds C1, C2, C3, and C4 to bFGF-supplemented experimental cultures led to an increase in neurospheres larger than 50 μm in diameter. The observed counts per mm^2^ were: for C1, 4.42 ± 0.92 (10 μM, n = 16, p < 0.001) and 3.45 ± 0.83 (100 μM, n = 17, p < 0.001); for C2, 0.55 ± 0.40 (10 μM, n = 17) and 0.55 ± 0.40 (100 μM, n = 18); for C3, 3.65 ± 1.10 (10 μM, n = 16, p < 0.01) and 2.70 ± 0.86 (100 μM, n = 17, p < 0.05); and for C4, 6.14 ± 1.20 (10 μM, n = 17, p < 0.001) and 0.34 ± 0.24 (100 μM, n = 18) (Figure 6b). The addition of C5 to the culture medium, at various tested concentrations, did not result in the formation of neurospheres with a diameter greater than 50 μm. Separately, for neurospheres smaller than 50 μm, higher concentrations of C3 led to a significant increase in their number (p < 0.01), consistent with previous observations of C3’s effect on these smaller neurospheres (Figure 6b).

Culturing NSCs with no added bFGF resulted in formation of primary adhesive neurospheres of various diameters in control cultures, namely, diameter of <50 μm - 46.14 ± 2.55 (n = 35) and >50 μm - 0.86 ± 0.28 (n = 35) per mm^2^ (Figures 6c,d). Addition of 10 μM C1 significantly decreased the number of neurospheres with a diameter of <50 μm - 27.99 ± 2.09 (n = 16) (p < 0.001) per mm2, while adding 100 μM C1 significantly increased the number of neurospheres to 61.22 ± 3.16 (n = 18) (p < 0.001) per mm^2^ (Figure 6c). Moreover, a significant difference (p < 0.001) in the number of neurospheres formed was observed when C1 of different concentrations was added: increased C1 concentration led to significantly greater number of neurospheres with a diameter of <50 μm (Figure 6c). On the other hand, both experimental concentrations, 10 μM and 100 μM, of C2 in the culture medium led to an increase in the number of neurospheres with a diameter of <50 μm: 76.53 ± 5.25 (n = 15) (p < 0.001) and 58.33 ± 3.50 (n = 16) (p < 0.001) per mm^2^, respectively (Figures 4d, 6c). Apart from that, increasing concentration of C2 led to a significant (p < 0.01) decrease in the number of neurospheres with a diameter of <50 μm, although this figure was substantially higher than in the control cultures (Figures 4, 6c). C3-C5 at concentrations of 10 μM and 100 μM contributed to a decrease in the number of neurospheres with a diameter of <50 μm: 33.43 ± 2.58 (n = 19) (p < 0.001), 27.81 ± 2.24 (n = 17) (p < 0.001), 5.71 ± 1.01 (n = 14) (p < 0.001) та 42.24 ± 2.88 (n = 17), 21.83 ± 2.67 (n = 18) (p < 0.001), 1.35 ± 0.83 (n = 16) (p < 0.001) per mm^2^, respectively (Figures 4f,h, 6c). Although when 100 μM C3 was added to the culture medium, a significant (p < 0.05) increase in the number of neurospheres of a given diameter was observed, compared to cultures cultivated with 10 μM C3, however, this indicator was still lower than the control (Figure 6c). Increasing the concentration of C5 led to a significant (p < 0.01) decrease in the number of neurospheres with a diameter of <50 μM (Figure 6c).

The addition of C1-C5 to the NSC culture medium also affected the formation of neurospheres larger than 50 μm in diameter. Specifically, C1-C3 at both 10 μM and 100 μM concentrations increased the number of these larger neurospheres: 1.23 ± 0.89 (n = 16), 3.68 ± 0.80 (n = 15) (p < 0.01), 2.58 ± 1.17 (n = 19) та 6.81 ± 1.47 (n = 18) (p < 0.001), 3.07 ± 0.92 (n = 16) (p < 0.05), 3.25 ± 0.80 (n = 17) (p < 0.05) per mm^2^, respectively (Figure 6d). Notably, a significant difference (p < 0.01) in the number of neurospheres (>50 μm) was observed with varying C1 concentrations; higher C1 concentrations resulted in a significantly greater number of these larger neurospheres (Figure 5d). Adding 10 μM C4 to the culture medium slightly increased the number of neurospheres >50 μm to 1.07 ± 0.46 per mm^2^ (n = 17). In comparison, 100 μM C4 led to a decrease to 0.67 ± 0.40 per mm^2^ (n = 18) (Figure 6d). Regardless of the presence or absence of bFGF, C5 at all tested concentrations inhibited the formation of neurospheres with a diameter exceeding 50 μm.

**FIGURE 7:**
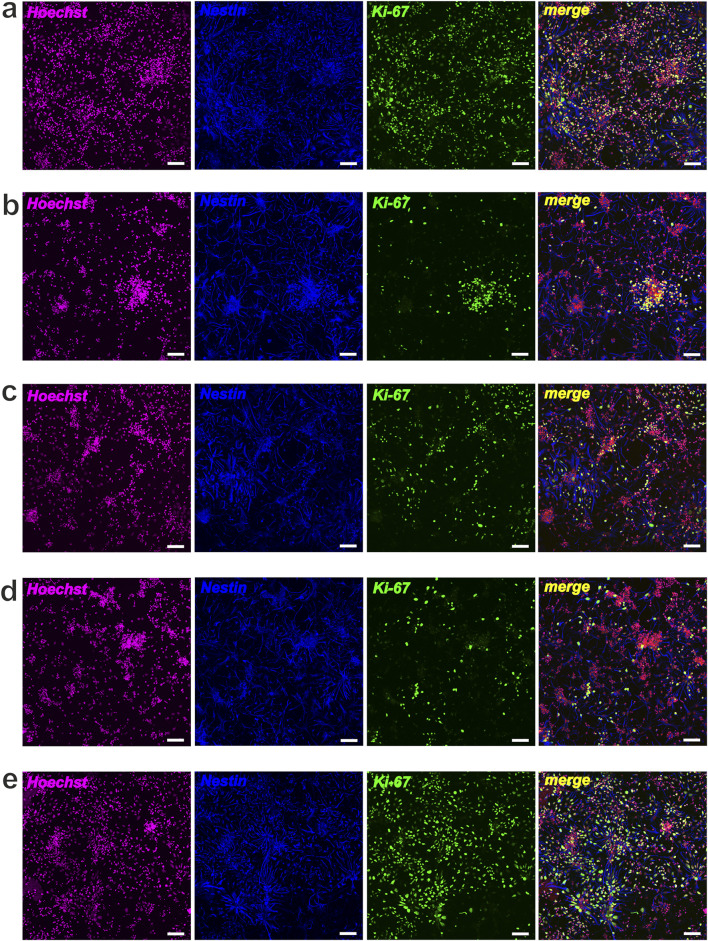
NSCs proliferative activity after cultivation (48 h) with Compounds (C1-C5). Representative confocal images of NSCs stained for Hoechst (magenta), Nestin (blue) and Ki-67 (green): **(a)** control, NSCs + bFGF; **(b)** NSCs + bFGF+10 μM C2; **(c)** NSCs + bFGF+100 μM C2; **(d)** control, NSCs w-o bFGF; **(e)** NSCs+10 μM C4; scale bars = 50 µm.

**FIGURE 8:**
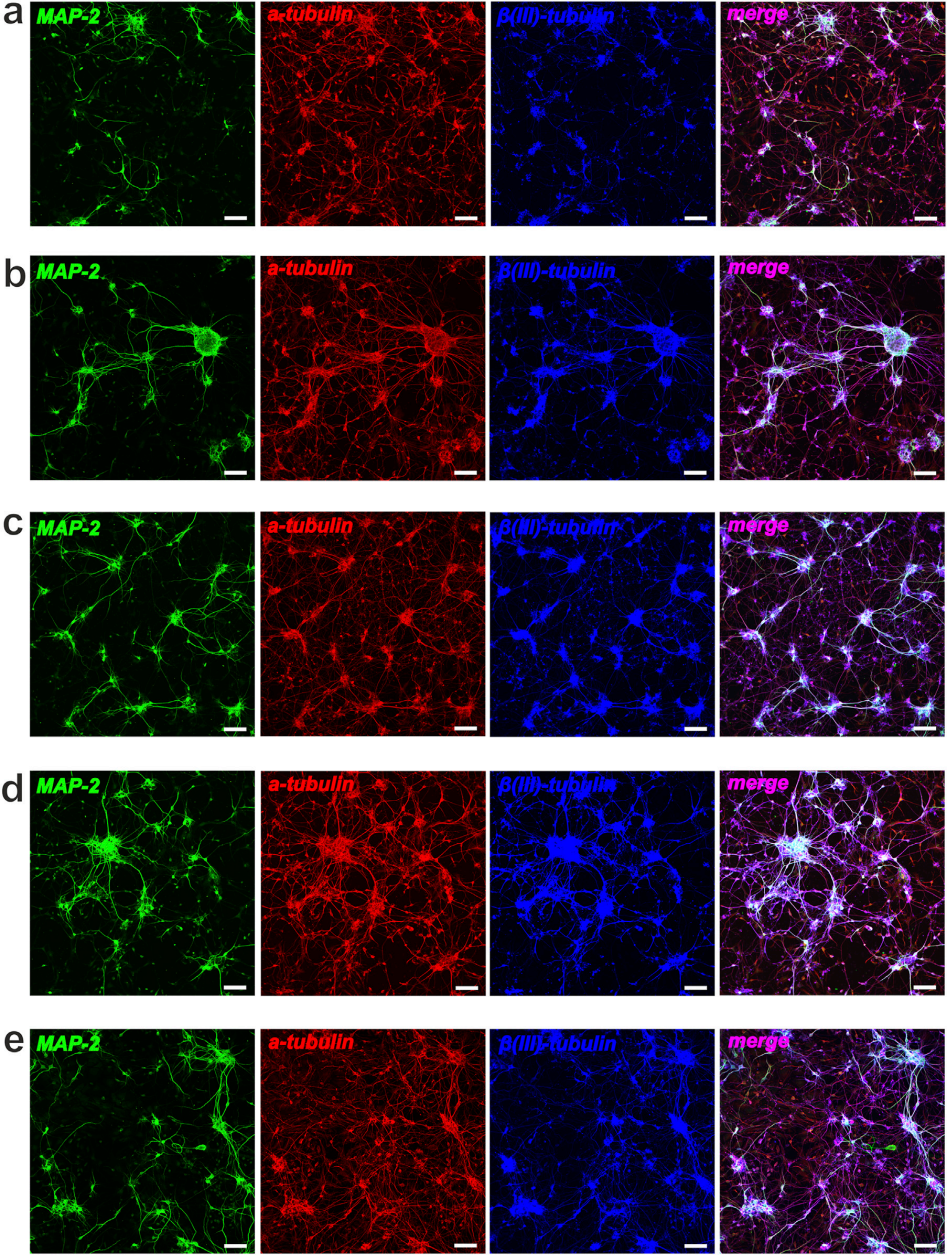
Neuronal differentiation of NSCs after cultivation (48 h) with Compounds (C1-C5). Representative confocal images of NSCs stained for MAP-2 (green), ɑ-tubulin (red) and β(III)-tubulin (blue): **(a)** control, NSCs + bFGF; **(b)** NSCs + bFGF+10 μM C2; **(c)** NSCs + bFGF+100 μM C2; **(d)** control, NSCs w-o bFGF; **(e)** NSCs+10 μM C4; scale bars = 50 µm.

In this study, we also performed immunocytochemical evaluation of NSCs cultured under different conditions. NSCs grown under standard conditions (with bFGF) maintained their undifferentiated state, as indicated by Nestin, and showed significant proliferative potential, as indicated by Ki-67 (Figure 7a). In these standard cultures (with bFGF), we also observed a small number of MAP-2-positive neurons and α- and β(III)-tubulin-positive elements of the neuroblast cytoskeleton, indicating the onset of differentiation in individual cells or within neurospheres (Figure 8a). In contrast, culturing NSCs without bFGF led to a significant decrease in Ki-67-positive cells (reduced proliferation) and a substantial increase in differentiation into neurons, as evidenced by the markers as mentioned above (Figures 7d, 8d).

Following the addition of C1–C4 at 10 μM and 100 μM, we observed a decrease in total cell number and a significant reduction in NSC proliferation, accompanied by an increase in cells expressing neuronal markers MAP-2, α-tubulin, and β(III)-tubulin (Figures 7a–c). Significant neuronal differentiation was also apparent within neurospheres. It is interesting to note that when cultivating NSCs (+bFGF) with C2 (at different concentrations), a decrease in proliferative activity according to the Ki-67 marker was observed, along with a significant increase in the number of neurospheres, which mainly contained differentiated cells - neurons (Figures 7b,c, 8b,c). Notably, in bFGF-free medium, 10 μM C4 significantly increased the number of proliferating NSCs compared to the control (Figure 7e). This indicates that C4 at this specific concentration can support NSC proliferation independently of exogenous bFGF. Conversely, the presence of C4 significantly inhibited both neuronal differentiation and neurosphere formation compared to the control cultures (Figure 8e).

In summary, short-term (48 h) culture with C1–C4 allows NSCs to maintain viability and other stem cell characteristics. These compounds were also found to affect NSC proliferation and the formation of primary adhesive neurospheres. Additionally, the presence of C1, C2, C3, and C4 led to noticeable qualitative changes in the α-tubulin and β(III)-tubulin components of the cellular cytoskeleton’s microtubules. Therefore, we conclude that C1–C4 directly influence NSC proliferation and differentiation into neurons without exhibiting cytotoxicity at the tested concentrations. In contrast, C5 was found to be toxic to NSC cultures at the concentrations used.

## Conclusion

Despite the binding site’s challenging geometry, we developed an effective high-throughput screening model using a sophisticated combination of structural and ligand-based approaches. The on-the-fly parameter tuning proved beneficial, allowing for rapid and accurate analysis of large databases and ensuring procedural reproducibility that accommodated changes at each iteration. Consequently, despite the lack of specific reference inhibitors, our molecular screening identified a set of compounds with potential activity. Structural analysis of αTAT1 complexes with experimentally validated ligands allowed us to propose three pharmacophore hypotheses for specific inhibition and to develop the most functional docking model for αTAT1. Importantly, short-term cultivation of neural stem cells with the novel molecules (C1, C2, C3, C4) demonstrated that the NSCs maintained their viability and other characteristic stem cell properties. We observed that C1, C2, C3, and C4 added to the culture medium influence NSC proliferation and the formation of primary adhesive neurospheres. Furthermore, these compounds induced qualitative and quantitative changes in the cytoskeletal microtubule proteins α-tubulin and β(III)-tubulin. Thus, our findings indicate that the new molecules (C1–C4) directly impact NSC proliferation and differentiation (especially towards neurons) and are not cytotoxic at the concentrations used. The obtained data will be used to refine the pharmacophore model for improved accuracy and to facilitate further screening of the Enamine (https://enamine.net.ua/) and Life Chemicals (https://lifechemicals.com) commercial databases.

This study contributes to a project aiming to identify indirect modulators of the nociceptive nervous system. Despite the separate development of a method for studying pain-related ion channels (Platonov et al., 2024a; Platonov et al., 2024b), the enhanced αTAT1 inhibitory activity suggests a future point of convergence for indirectly blocking the pain cascade.

## Data Availability

The original contributions presented in the study are included in the article/supplementary material, further inquiries can be directed to the corresponding author.
